# A subwavelength spot and a three-dimensional optical trap formed by a single planar element with azimuthal light

**DOI:** 10.1038/s41598-017-07810-8

**Published:** 2017-08-07

**Authors:** Jian Guan, Jie Lin, Yuan Ma, Jiubin Tan, Peng Jin

**Affiliations:** 1 Institute of Ultra-Precision Optoelectronic Instrument Engineering, Harbin Institute of Technology, Harbin, 150080 China; 2 Department of Electrical and Computer Engineering, Dalhousie University, Halifax, Nova Scotia B3J 1Z1 Canada

## Abstract

The generation of subwavelength spots smaller than the Abbe diffraction limit has attracted great interest due to the various applications in many fields, such as high-density optical data storage and particle manipulation. Planar optics that can miniaturize conventional refractive optics have become increasingly attractive. In this work, we first formed a subwavelength bright spot and a three-dimensional optical trap under the illumination of an azimuthally polarized (AP) beam by only a single planar element, a spiral zone plate (SZP). Initially, the SZP was proposed as a computer-generated hologram to generate optical phase singularities. However, the SZP in this work was used to focus and modulate the incident AP beam with a vortex phase simultaneously. Therefore, no additional vortex phase modulating element was introduced in our method. The SZP has an ultra-long focal length of 250*λ* for a numerical aperture (NA) of 0.95 and an incident wavelength of 632.8 nm. The generated spot is purely transversely polarized with a lateral full width at half maximum (FWHM) of 0.43*λ* beyond the diffraction limit of 0.54*λ*. The generated focal field formed a stable optical trap for a Rayleigh dielectric particle in three dimensions.

## Introduction

Subwavelength spots smaller than the Abbe diffraction limit have attracted great interest due to their wide applications in photolithography^1, 2^, high-density optical data storage^3^, super-resolution imaging^4, 5^ and particle manipulation^6, 7^. The near-field optical technologies can overcome the Abbe diffraction limit and achieve super-resolution spots^8^. However, the extremely short working distances limit the industrial applications of these near-field methods. In the far-field, radially polarized (RP) beams have been focused into subwavelength spots and light needles by high numerical aperture (NA) systems^9–15^. However, theoretical studies showed that an azimuthally polarized (AP) beam could only be focused to a doughnut-shaped spot by an objective^16^. Recently, an AP beam has been focused into a bright spot when it was modulated by a vortex 0–2*π* phase plate^17–19^. Furthermore, the generated focal spot was smaller than that produced by focusing a RP beam with the same aplanatic lens. However, the previously mentioned subwavelength spots generated by RP or AP beams were achieved by conventional refractive lenses. For making optical systems to be more easily integrated, planar optical elements have been widely investigated as a good alternative to conventional refractive optical elements because they are smaller and lighter^20, 21^. Recently, a planar metalens was proposed to focus an AP beam into a subwavelength light needle^22^. This metalens cannot modulate the incident AP beam with a vortex 0–2*π* phase. Therefore, a holographic fork grating, which works as a vortex 0–2*π* phase modulating element, was essential in this work to generate the centre bright focal field.

In this work, we first formed a subwavelength bright spot and a three-dimensional optical trap under the illumination of an AP beam by only a single planar element, a spiral zone plate (SZP). Initially, the SZP was proposed as a computer-generated hologram to generate optical phase singularities^23^. However, the effects of the SZP in our work are focusing and modulating the incident AP beam with a vortex phase simultaneously. Therefore, no additional vortex phase modulating element is needed and the proposed system is more compact. The SZP has a larger apodization factor than an aplanatic lens. Thus, the generated spot is smaller than the one produced using an aplanatic lens with a vortex 0–2*π* phase plate. Moreover, the focal length of the SZP is 250*λ* for a numerical aperture (NA) of 0.95 and a wavelength of 632.8 nm. This ultra-long focal length ensures sufficient space between the SZP and the specimen, which is significant for practical industrial applications^22^. Meanwhile, the generated focal field forms a stable three-dimensional optical trap for a Rayleigh dielectric particle in air. Previously, AP beams could only form the focal fields with hollow cores directly and small particles could not be trapped on the central axis by AP beams.

## Theory and Method

**Figure 1:**
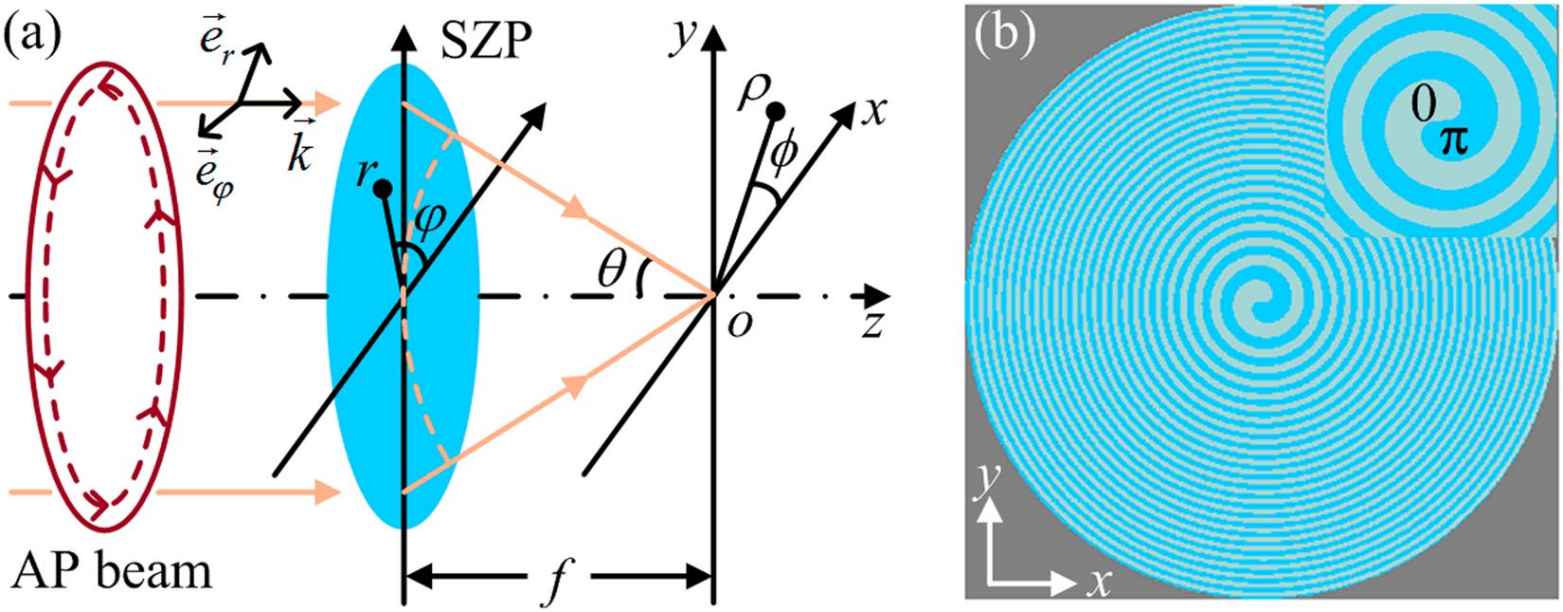
Schematic of forming a subwavelength spot and a three-dimensional optical trap by a single planar element with AP light. (**a**) System layout, and (**b**) phase structure of the SZP with *p* = 1 and *N*_max_ = 24. Inset is the enlarged image of the SZP centre region.

The schematic of forming a subwavelength spot and a three-dimensional optical trap by a single planar element with AP light is shown in Fig. 1. The incident beam propagates along the *z*-axis, and the direction is denoted by the wave vector $\overrightarrow{k}$. ${\overrightarrow{e}}_{r}$ and ${\overrightarrow{e}}_{\phi }$ are the unit vectors of the radial and azimuthal directions, respectively. The focal plane of the SZP is at *z* = 0.

The transmittance function of the SZP with a high NA can be expressed as

1 $${{\rm{S}}{\rm{Z}}{\rm{P}}}_{p}(r,\phi )=\exp (ip\phi )\exp (-2\pi i\frac{\sqrt{{f}^{2}+{r}^{2}}-f}{\lambda }),$$

where *r* and *φ* are the polar coordinates of the SZP. *p* is the topological charge. *f* is the focal length. *λ* is the wavelength in the medium of the focal volume with a refractive index *n*_1_. The exponential term with *φ* represents the vortex phase. When the topological charge *p* is 1, the SZP implements a vortex 0–2*π* phase modulation. The exponential term, including *r* and *f*, represents a focusing phase. Clearly, the SZP can focus and encode the incident beam with a vortex phase simultaneously. As mentioned in the introduction, an AP beam could been focused into a bright spot by applying a vortex 0–2*π* phase modulation^17–19^. Therefore, a bright spot can be generated by only a single SZP under the illumination of AP light. Additional vortex phase modulating elements are no longer needed. The proposed system is smaller, lighter and more easily integrated. To achieve a more feasible fabrication, the practical SZP is a binary planar diffractive element. Compared with a binary amplitude SZP, a binary phase SZP can achieve the higher energy efficiency. Thus, the phase SZP is chosen in this work and it can be expressed as

2 $${{\rm{B}}{\rm{S}}{\rm{Z}}{\rm{P}}}_{p}(r,\phi )=\{\begin{array}{cc}1 & 2n\pi \le p\phi -\frac{2\pi (\sqrt{{f}^{2}+{r}^{2}}-f)}{\lambda } < (2n+1)\pi \\ -1 & (2n+1)\pi \le p\phi -\frac{2\pi (\sqrt{{f}^{2}+{r}^{2}}-f)}{\lambda } < (2n+2)\pi ,\end{array}$$

where *n* is an integer variable. When *p* is a nonzero integer, the SZP is referred to as the *p*th-order SZP. For the first-order SZP, *n* = 0, −1, −2, …, −*N*_max_, and *N*_max_ is restricted by the aperture of the SZP. The phase structure of the first-order SZP with *N*_max_ = 24 is shown in Fig. 1(b), and it is planar spiral and no longer circularly symmetrical. When the topological charge *p* is zero, the SZP becomes a well-known Fresnel zone plate (FZP)^24^. For the corresponding FZP, *n* = −1, −2, …, −(*N*_max_ + 1), and 2*N*_max_ + 2 is the ordinal number of the outermost belt.

According to the vector diffraction theory, the electric field components near the focus of a first-order SZP illuminated by an AP beam can be written as^16, 25^.

3 $$[\begin{array}{c}{E}_{x}(\rho ,\varphi ,z)\\ {E}_{y}(\rho ,\varphi ,z)\\ {E}_{z}(\rho ,\varphi ,z)\end{array}]=\frac{-ik{l}_{0}\,f}{2\pi }{\int }_{0}^{\alpha }{\int }_{0}^{2\pi }F\,\sin \,\theta P(\theta ){{\rm{B}}{\rm{S}}{\rm{Z}}{\rm{P}}}_{+1}(r,\phi )[\begin{array}{c}-\,\sin \,\phi \\ \cos \,\phi \\ 0\end{array}]{e}^{i{\rm{\Phi }}(\theta )}{e}^{ik[z\cos \theta +\rho \sin \theta \cos (\phi -\varphi )]}{\rm{d}}\theta {\rm{d}}\phi ,$$

where *k* is the wave number in the medium of the focal volume. *l*_0_ is the relative amplitude of the incident AP beam, and *α* = arcsin(NA/*n*_1_) is the maximal convergence angle. The longitudinally polarized component is null, and the focal electric field is completely transversely polarized. Because the SZP obeys the Helmholtz condition of *r* = *f* tan*θ* ^7^, the corresponding apodization factor *F* of the SZP is cos^−3/2^ *θ* ^26^. *P*(*θ*) is the amplitude of the incident field, and the Bessel-Gaussian beam is employed in this work, which is expressed as

4 $$P(\theta )=\exp [-{\beta }^{2}{(\frac{\tan \theta }{\tan \alpha })}^{2}]{J}_{1}(2\beta \frac{\tan \,\theta }{\tan \,\alpha }),$$

where *β* is the ratio between the pupil radius and the beam waist, and it is 0.5 in this work^16^. *J*_1_(*x*) is the first kind of the first-order Bessel function. Φ(*θ*) is the phase difference between the converging wave front and the ideal spherical wave, which can be written as

5 $${\rm{\Phi }}(\theta )=kf(1-1/\cos \,\theta ).$$

According to the geometrical relationship between the Cartesian coordinates and the cylindrical coordinates, the radially and azimuthally polarized components of the focal electric field can be expressed as

6 $$\{\begin{array}{c}{E}_{\rho }={E}_{x}\,\cos \,\varphi +{E}_{y}\,\sin \,\varphi ,\\ {E}_{\varphi }=-{E}_{x}\,\sin \,\varphi +{E}_{y}\,\cos \,\varphi .\end{array}$$

The magnetic field components *H*_*z*_, *H*_*y*_ and *H*_*z*_ can be easily calculated based on Maxwell’s equations by replacing [−sin*φ*, −cos*φ*, 0]′ on the right side of equation (3) with *n*_1_ *ε*_0_ *c*[−cos*φ*cos*θ*, −sin*φ*cos*θ*, sin*θ*]′. *ε*_0_ is the vacuum dielectric constant and *c* is the speed of light in vacuum.

## Results

### Subwavelength spot

**Figure 2:**
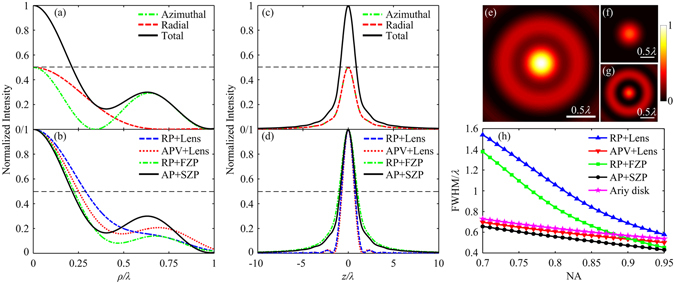
Normalized electric energy density in the focal volume. (**a**,**b**) Electric energy density profiles along the radial direction on the focal plane. (**c**,**d**) Electric energy density profiles along the optical axis *z*. (**a**) and (**c**) are for the total intensity, radial and azimuthal components of the spot generated by the SZP. (**b**) and (**d**) are for the different polarized beams and focusing systems. (**e**–**g**) Two-dimensional field distributions of the total intensity, radial and azimuthal components on the focal plane for the spot generated by the SZP. (**h**) Lateral FWHMs of the spots generated by different focusing systems versus the NA.

Numerical calculation is executed to validate the proposed method of generating a subwavelength spot by a single planar element under the illumination of AP light. The incident wavelength in vacuum is 632.8 nm, and the refractive index of the medium *n*_1_ is 1. The NA and *N*_max_ of the SZP are designed as 0.95 and 549, respectively. The radius of the SZP is 480.75 μm. The narrowest linewidth of the SZP is 300 nm. The designed SZP can be fabricated using electron beam lithography. The focal length of the SZP is 158.02 μm, which is approximately 250 times the wavelength. Consequently, the focal spot is located in the far-field, and the effect of the evanescent wave can be completely ignored. The vector diffraction theory is sufficiently accurate for the calculation. The normalized electric energy density on the focal plane of the first-order SZP illuminated by an AP Bessel-Gaussian beam is shown in Fig. 2(a) and (e). A bright focal spot is achieved by the single planar element because the vortex 0–2*π* phase introduced by the SZP converts the deconstructive interference between the transverse focal components to the constructive interference^18^. Although the SZP is spiral and not circularly symmetric, the generated spot is circularly symmetric, which always has more practical applications than non-circular spots. As shown in Fig. 2(a) and (f), an evident radial component appears in the focal volume, which does not exist in the incident light. The radial component only has a centre main lobe, while the azimuthal component has a remarkable annular side lobe. Consequently, the side lobe of the focal spot is dominated by the azimuthal component. However, because the radial component prominently enhances the main lobe and does not contribute to the side lobe, the side lobe intensity becomes tiny by contrast. The peak intensity of the side lobe is 30% of the main lobe intensity. Moreover, the radial and azimuthal components on the optical axis *z* are identical, as shown in Fig. 2(c). This means that the radial and azimuthal components contribute equally to the total electric energy density on the optical axis.

The lateral FWHMs of the radial, azimuthal components and the total focal spot generated by the designed SZP are 0.6*λ*, 0.34*λ* and 0.43*λ*, respectively. The generated spot is smaller than the Abbe diffraction limit or the Airy disk with a FWHM of 0.51*λ*/NA, namely, 0.54*λ*. Additionally, the axial FWHM of the focal spot is 1.8*λ*, and the corresponding volume of the spot is 1.39*λ* ^3^, which is fairly small for high-density optical data storage^3^. To further clarify the characteristics of the generated spot, the normalized electric energy density distributions along the radial direction on the focal plane for different polarized beams and focusing systems are shown in Fig. 2(b). The APV beam is the azimuthally polarized beam modulated by an additional vortex 0–2*π* phase element. The lens is an aplanatic objective that obeys the sine condition. All incident beams are the same Bessel-Gaussian beams. The tangent terms in equation (4) are revised as the corresponding sine terms for the calculation of the lens. The FZP has the same NA and *N*_max_ as the SZP and can implement the identical function as the SZP except for the vortex phase modulation. The FZP has 1100 concentric belts. The NAs of all focusing systems are 0.95. The lateral FWHMs of RP + Lens, APV + Lens and RP + FZP are 0.578*λ*, 0.5*λ* and 0.452*λ*, respectively. Clearly, the focal spot generated by the proposed method of AP + SZP is the smallest. The lateral FWHM of the generated spot, 0.43*λ*, is reduced by 14% compared to the 0.5*λ* of APV + Lens because the planar SZP has a higher apodization factor than an aplanatic refractive lens for the same NA. The normalized electric energy density distributions along the optical axis *z* for different polarized beams and focusing systems are shown in Fig. 2(d). The axial FWHMs of RP + Lens, APV + Lens and RP + FZP are 1.5*λ*, 1.4*λ* and 2.1*λ*, respectively. The spot generated by the proposed method of AP + SZP is longer than that by APV + Lens, and the spot generated by RP + FZP is longer than that by RP + Lens. These results indicate that a planar element can slightly elongate the generated spot compared with an aplanatic objective under the same condition. Moreover, by comparing the axial intensities of AP + SZP and RP + FZP or APV + Lens and RP + Lens, one can find that the spots generated by AP or APV beams are shorter than those by RP beams when the focusing systems have the same apodization factor.

The lateral FWHMs of the spots generated by different focusing systems versus the NA are shown in Fig. 2(h). The markers on the lines are the calculated points. All SZPs under different NAs have the same focal length of 158.02 μm. The maximal radii of these SZPs change with the NA. Under the same NA, the FZP has the same *N*_max_ as the SZP. The FWHMs of the Airy disks for different NAs are also plotted in Fig. 2(h). The SZP and FZP have the same apodization factor, which is larger than that of an aplanatic lens. Therefore, the spots generated by the proposed method of AP + SZP are always smaller than those by APV + Lens, and the spots generated by RP + FZP are always smaller than those by RP + Lens. Meanwhile, for the NA from 0.7 to 0.95 the spots generated by AP or APV beams are always smaller than those generated by RP beams when the focusing systems have the same apodization factor. This confirms that a smaller spot can be generated when the incident light is an AP or APV beam instead of a RP beam^17^. Moreover, it is clear that the focal spots generated by the proposed method of AP + SZP are always the smallest and beyond the diffraction limit. These results demonstrate the proposed method of generating a subwavelength spot under the illumination of AP light beam by only a single planar element. Meanwhile, the generated subwavelength spot is insensitive to the fabrication errors of the designed SZP. The calculated results in the supplementary materials indicate that the lateral FWHM of the generated spot only varies by 1.7% for the maximum radius deviation of 50 nm. For a commercial electron beam lithography system, the tolerance of 50 nm is relatively easy to achieve.

As previously mentioned, the generated focal electric field is purely transversely polarized. The detailed polarization properties can be further investigated in term of the Stokes parameters. The azimuthal angle *ψ* and the ellipticity tan*χ* of the local polarization ellipse can be calculated using

7 $$\{\begin{array}{c}\tan \,2\psi =({E}_{x}{E}_{y}^{\ast }+{E}_{y}{E}_{x}^{\ast })/({E}_{x}{E}_{x}^{\ast }-{E}_{y}{E}_{y}^{\ast })\\ \sin \,2\chi =i({E}_{x}{E}_{y}^{\ast }-{E}_{y}{E}_{x}^{\ast })/({E}_{x}{E}_{x}^{\ast }+{E}_{y}{E}_{y}^{\ast }),\end{array}$$

where ${E}_{x}^{\ast }$ and ${E}_{y}^{\ast }$ are the conjugate complex of *E*_*x*_ and *E*_*y*_.

**Figure 3:**
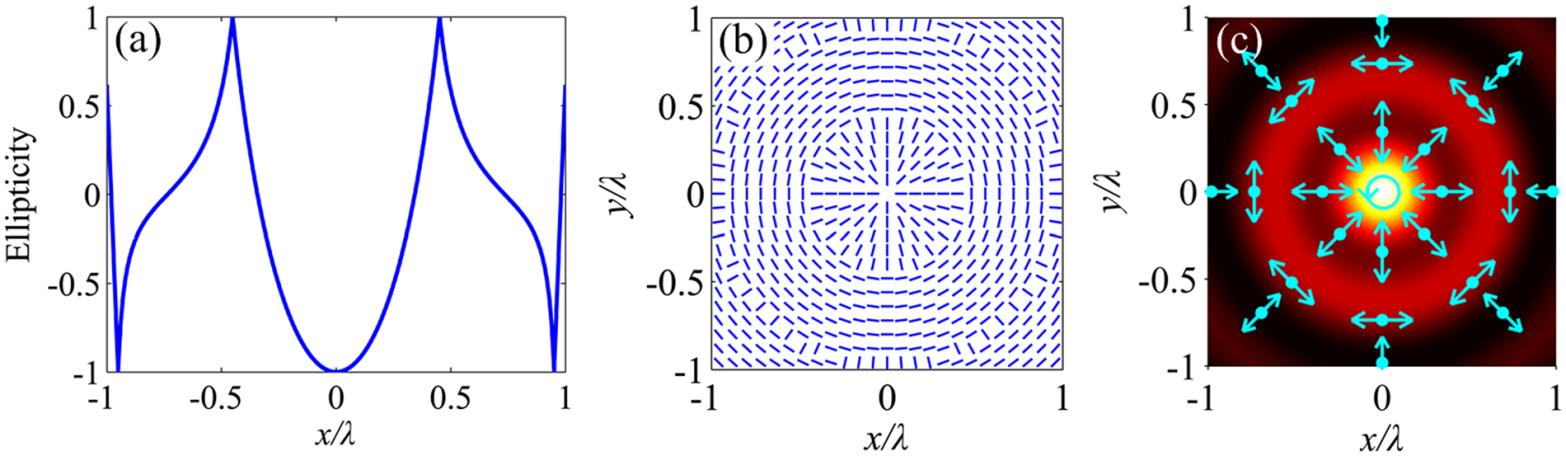
Polarization of the electric field on the focal plane of the first-order SZP illuminated by an AP Bessel-Gaussian beam. (**a**) Ellipticities tan*χ* of the local polarization ellipses along the *x-*axis. (**b**) Orientations of the long axes of the local polarization ellipses on the focal plane. (**c**) Polarization directions at particular positions on the focal plane.

The spatially varying polarization feature of the electric field on the focal plane is shown in Fig. 3. The ellipticity is −1 at the spot centre of *x* = 0, which is shown in Fig. 3(a). Therefore, the generated focal electric field is strictly circularly polarized at the spot centre. At most other positions, the focal electric fields are elliptically polarized, and the long axes of the local polarization ellipses are either radial or azimuthal, which are denoted by the short lines in Fig. 3(b). The particular positions where the ellipticities are zero or ± 1 should be noted. For *ρ* = 0.343*λ*, the ellipticity becomes zero first, and the focal electric field is radially polarized where the azimuthal component equals zero, as shown in Fig. 3(a) and Fig. 2(a). For *ρ* = 0.735*λ*, the ellipticity becomes zero second where the radial component equals zero. Therefore, the focal electric field is purely azimuthally polarized at *ρ* = 0.735*λ*. The focal electric field is either radially or azimuthally polarized at the positions where the ellipticities are zero. The arrows in Fig. 3(c) represent the polarization directions at the particular positions denoted by the midpoints of the arrows. The circular polarization at the centre is also marked. Additionally, the ellipticities are ± 1 at the positions where the intensities of the radial and azimuthal components are equal. These positions form the boundaries where the orientations of the long axes of the local polarization ellipses change, as shown in Fig. 3(b). At theses boundaries, the polarization is circular, and the azimuthal angle is undefined.

**Figure 4:**
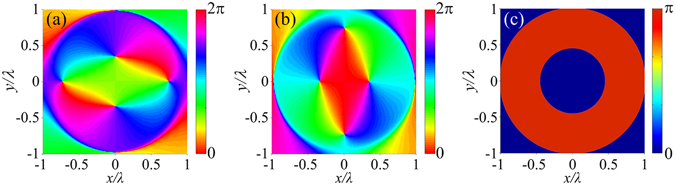
Phase distribution of the electric field on the focal plane of the first-order SZP illuminated by an AP Bessel-Gaussian beam. (**a**) Argument of the *x*-component of the electric vector. (**b**) Argument of the *y*-component of the electric vector. (**c**) Phase of the total electric field according to Pancharatnam’s definition.

To map the phase distribution of the electric field on the focal plane with space-variant polarization, Pancharatnam’s definition for phase, $\gamma =\arg \langle \overrightarrow{E}({\overrightarrow{r}}_{1})|\overrightarrow{E}({\overrightarrow{r}}_{2})\rangle$, is applied^27^. $\langle \overrightarrow{E}({\overrightarrow{r}}_{1})|\overrightarrow{E}({\overrightarrow{r}}_{2})\rangle$ is the inner product of $\overrightarrow{E}(\overrightarrow{{r}_{1}})$ and the conjugate vector of $\overrightarrow{E}(\overrightarrow{{r}_{2}})$. ${\overrightarrow{r}}_{1}$ is the position vector of an observation point on the focal plane with coordinates (*ρ*, *ϕ*). ${\overrightarrow{r}}_{2}$ is the position vector of the reference point. In the calculation, the spot centre with coordinates (0, *ϕ*) is chosen as the reference point. Therefore, *γ* represents the phase difference between the observation point and the spot centre. Because the polar angle *ϕ* of the spot centre is uncertain, the radial and azimuthal components of the electric field cannot be used to calculate the phase distribution. Alternatively, the *x*- and *y-*components are used. The phase distribution of the total electric field on the focal plane can be expressed as $\gamma (\rho ,\varphi )={\rm{\arg }}[{E}_{x}(\rho ,\varphi ){E}_{x}^{\ast }(0,\varphi )+{E}_{y}(\rho ,\varphi ){E}_{y}^{\ast }(0,\varphi )]$. The argument distributions of the *x*- and *y-*components of the generated electric vectors on the focal plane are intricate, as shown in Fig. 4(a) and (b). However, the phase of the total focused electric field is very concise and only includes 0 and *π*, as shown in Fig. 4(c). There is no phase singularity at the focus, which is consistent with the result that the spot is bright at the centre.

### Three-dimensional optical trap

The generated focal field forms a stable three-dimensional optical trap for a Rayleigh dielectric particle, whose radius *a* is much smaller than the wavelength, usually *a* < *λ*/20. For such a Rayleigh particle, the optical forces exerted on it can be accurately calculated by^28, 29^

8 $$\overrightarrow{F}=\frac{1}{4}\mathrm{Re}(\sigma ){\boldsymbol{\nabla }}{|\vec{E}|}^{2}+\frac{{k}_{0}}{{\varepsilon }_{0}c}\text{Im}(\sigma )\langle \vec{S}\rangle +\frac{{k}_{0}c}{{\varepsilon }_{0}}\text{Im}(\sigma ){\boldsymbol{\nabla }}\times \langle {\vec{L}}_{s}\rangle ,$$

where *σ* is given by $\sigma =\frac{{\sigma }_{0}}{1-i{\sigma }_{0}\,{k}_{0}^{3}/(6\pi {\varepsilon }_{0})}$ and ${\sigma }_{0}=4\pi {\varepsilon }_{0}{a}^{3}\frac{{n}_{2}^{2}/{n}_{1}^{2}-1}{{n}_{2}^{2}/{n}_{1}^{2}+2}$. Here, *n*_2_ is the refractive index of the Rayleigh particle and *k*_0_ is the wave number in vacuum. *n*_1_ is the aforementioned refractive index of the medium and equals 1. $\langle \overrightarrow{S}\rangle$ is the time averaged Poynting vector given by ${\rm{R}}{\rm{e}}\,(\overrightarrow{E}\times {\overrightarrow{H}}^{\ast })/2$, and $\langle \overrightarrow{{L}_{s}}\rangle$ is given by $\frac{{\varepsilon }_{0}}{4i{k}_{0}c}(\overrightarrow{E}\times {\overrightarrow{E}}^{\ast })$. The first term in equation (8) is the gradient force that can trap a small particle^28, 30^. The second term is the radiation pressure that may push the particle away from the focus, while the third term is the spin density force^29^.

**Figure 5:**
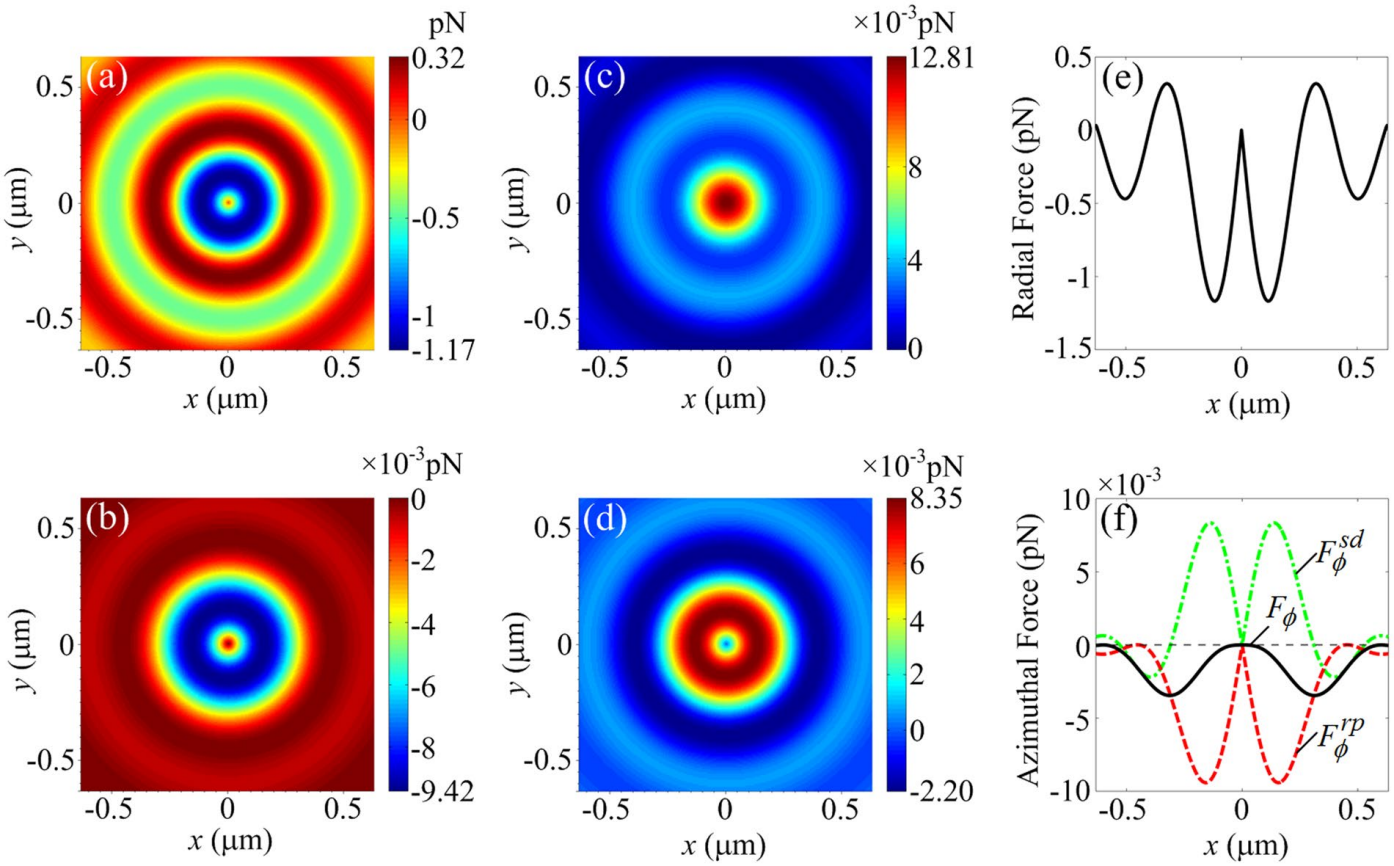
Optical forces on a 30 nm (radius) dielectric particle with *n*_2_ = 1.6 located on the focal plane. (**a**) Radial gradient force. (**b**) and (**c**) Azimuthal and axial radiation pressures. (**d**) Azimuthal spin density force. (**e**) and (**f**) Total radial and azimuthal optical forces along the *x*-axis.

The optical forces exerted on a Rayleigh dielectric particle located on the focal plane are shown in Fig. 5. The parameters for the calculation are taken as *a* = 30 nm and *n*_2_ = 1.6. A typical laser power for an optical trap of 1 W is employed^31^. The direction of the force is denoted in cylindrical coordinates. On the focal plane, the azimuthal and axial gradient forces, the radial radiation pressure, the radial and axial spin density forces are all null. The radial gradient force ranges from −1.17 pN to 0.32 pN, as shown in Fig. 5(a), which forms the total radial optical force on the focal plane. The azimuthal radiation pressure ranges from −9.42 × 10^−3^ pN to 0, and the negative sign expresses that the direction of the force is clockwise in Fig. 5(b). The axial optical force on the focal plane is completely formed by the axial radiation pressure, as shown in Fig. 5(c). It points in the positive direction of the *z-*axis, and the maximum is approximately 12.81 × 10^−3^ pN. The azimuthal spin density force ranges from −2.20 × 10^−3^ pN to 8.35 × 10^−3^ pN, as shown in Fig. 5(d), which is induced by the curl of the spin angular momentum of the light field^29^. The previous results have indicated that the generated focal field possesses spin angular momentum because the focal spot is circularly polarized at the centre and elliptically polarized at most other positions. The total radial optical force along the *x*-axis on the focal plane is shown in Fig. 5(e). It is null at the spot centre and negative around the centre, which means that the total radial optical force points to the focus. The total azimuthal optical force *F*_*ϕ*_ along the *x*-axis on the focal plane is shown in Fig. 5(f). ${F}_{\varphi }^{rp}$ and ${F}_{\varphi }^{sd}$ are the azimuthal radiation pressure and the azimuthal spin density force, respectively. At the spot centre, the azimuthal spin density force counteracts the azimuthal radiation pressure, where the total azimuthal optical force is exactly zero. Moreover, Fig. 5(f) shows that a small area around the spot centre is formed where the total azimuthal optical force is negligible. This area is larger than the size of the nanoparticle. Therefore, the particle will not spin at the focus. Clearly, by combining the results of the total radial and azimuthal optical forces on the focal plane, a transverse trap for the dielectric particle at the focus is formed. By contrast, for a particle with a refractive index higher than the medium, the transverse trap generated by directly focusing an AP beam can only confine the particle to an annular region at the focus rather than to a stable point^32^.

**Figure 6:**
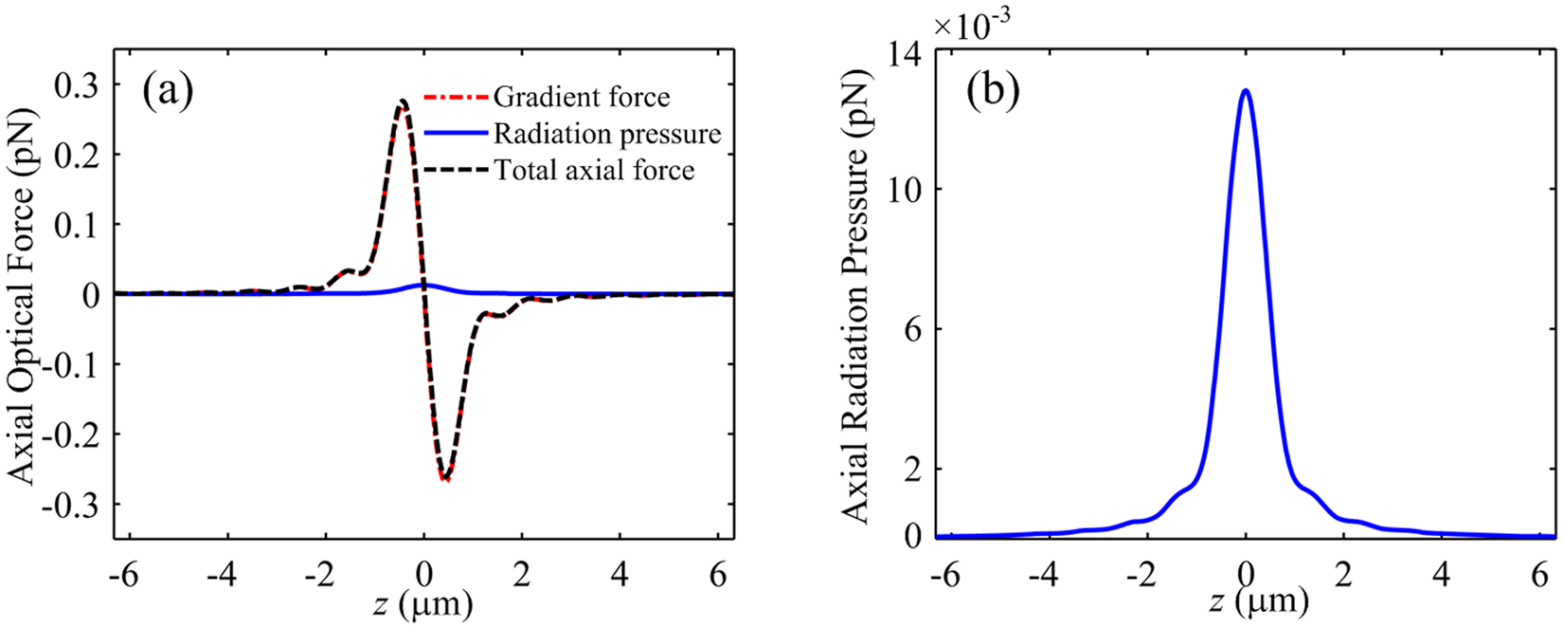
Axial optical forces on a 30 nm (radius) dielectric particle with *n*_2_ = 1.6 located on the optical axis *z*. (**a**) Total axial optical force. (**b**) Axial radiation pressure.

The axial optical forces on the particle located on the optical axis are shown in Fig. 6. The axial spin density force is null. Therefore, the total axial optical force is the sum of the axial gradient force and the axial radiation pressure, as shown in Fig. 6(a). The maxima of the axial gradient force and the axial radiation pressure are 0.27 pN and 12.81 × 10^−3^ pN, respectively. By contrast, the axial radiation pressure, which is plotted in Fig. 6(b), is so weak that it can be neglected. An axial optical trap is formed for the Rayleigh dielectric particle. It is completely different from the optical trap generated by directly focusing an AP beam, which cannot form an axial trap because the focal field is hollow on the optical axis.

The lateral and axial optical forces formed a three-dimensional optical trap for the Rayleigh dielectric particle. However, for Rayleigh scattering particles, Brownian motion can strongly affect the trapping. For a stable optical trap, the potential well of the gradient force needs to be much larger than the kinetic energy of the particle. The criterion is ${R}_{{\rm{thermal}}}={e}^{-{U}_{{\rm{\max }}}/{k}_{{\rm{B}}}T}\ll 1$, where *k*_B_ is the Boltzmann constant, and the maximal depth of the potential well is ${U}_{max}=\pi {\varepsilon }_{0}{n}_{1}^{2}{a}^{3}|\frac{{n}_{2}^{2}/{n}_{1}^{2}-1}{{n}_{2}^{2}/{n}_{1}^{2}+2}|{|\overrightarrow{E}|}_{max}^{2}$ ^33^. When the temperature *T* is 300 K, *R*_thermal_ for the nanoparticle with a radius of 30 nm is 8.14 × 10^−24^. Therefore, the generated three-dimensional optical trap is stable and will not be destroyed by Brownian motion.

## Discussion

We first formed a subwavelength bright spot and a three-dimensional optical trap under the illumination of an AP beam by only a single planar element, a spiral zone plate (SZP). The method does not involve any additional vortex 0–2*π* phase element or conventional refractive lens. Therefore, the proposed system is more compact. Moreover, if the photonic-crystal semiconductor laser that can directly output an AP Bessel-Gaussian beam is applied in our method, the proposed system can be further miniaturized^34^. When the SZP is directly integrated into the semiconductor laser, the laser can directly output a subwavelength focal spot. For the NA of 0.95 and the wavelength of 632.8 nm, the SZP has an ultra-long focal length of 250*λ*. There is sufficient space between the SZP and the specimen, and various practical industrial applications can be satisfied. The lateral FWHM of the generated spot is 0.43*λ*, namely, 272 nm, which is reduced by 14% compared with that of using an aplanatic lens to focus the AP beam with a vortex 0–2*π* phase. The explanation for this phenomenon is that the SZP has a larger apodization factor than an aplanatic lens. The axial FWHM of the focal spot is 1.8*λ*, and the corresponding volume of the spot is 1.39*λ* ^3^. Consequently, the generated spot is small enough in three dimensions and can be used for high-density optical data storage^3^. Furthermore, the generated focal electric field is circularly polarized at the spot centre and elliptically polarized at most other locations. The phase of the total focused electric field is binary variant on the focal plane, either 0 or *π*. Meanwhile, there is no phase singularity at the spot centre. For a Rayleigh dielectric particle with a radius of 30 nm, a stable three-dimensional optical trap was for the first time directly formed by an AP beam. Moreover, the optical trap can stably confine the particle to a point rather than to a region, and the particle will not spin in the optical trap. The generated focal field can be applied in high-density optical data storage and nanoparticle trapping. The proposed method will be doubtlessly beneficial for realizing functional flat optics.

## Electronic supplementary material

Supplementary Information
