# Supplementary material for: A subwavelength spot and a three-dimensional optical trap formed by a single planar element with azimuthal light
Source: Sci Rep. 2017 Aug 7;7:7380. doi: 10.1038/s41598-017-07810-8 (PMC5547119; doi:10.1038/s41598-017-07810-8)
Supplement: Supplementary file 1 — Supplementary Information [file 41598_2017_7810_MOESM1_ESM.pdf]

A subwavelength spot and a three-dimensional optical trap formed by a single planar element with azimuthal light

Jian Guan[1,+], Jie Lin[1,+,*], Yuan Ma[2], Jiubin Tan[1], and Peng Jin[1,*]

[1]Institute of Ultra-Precision Optoelectronic Instrument Engineering, Harbin Institute of Technology, Harbin 150080, China
[2]Department of Electrical and Computer Engineering, Dalhousie University, Halifax, Nova Scotia B3J 1Z1, Canada
[*]Corresponding author: linjie@hit.edu.cn (J.L.) and P.Jin@hit.edu.cn (P.J.)
[+]These authors contributed equally to this work.

Supplementary

The fabrication errors will affect the performance of the SZP, especially the radius fabrication deviation. In this supplementary, the influence of the radius fabrication deviation on the spot size is investigated.

For a circularly symmetrical device such as a FZP, the radius fabrication deviation will change the widths of the concentric belts. The influence of the radius deviation can be investigated by randomly changing the belt widths. However, the SZP is planar spiral and no longer circularly symmetrical. Consequently, the influence of the radius deviation of the SZP cannot be directly researched by this method.

We introduce the radius deviation into the calculation for the SZP by two different modes. The transmittance function of the *p*th-order phase SZP is expressed as equation (2) in the main body of the article. The first deviation introduction mode is implemented by adding a random radius deviation to the radial coordinate r in equation (2) of every calculated sample point of the SZP. The added random radius deviation of every sample point obeys the uniform distribution with $\pm\Delta r_{max}$, where Δr_{max} is the maximum radius deviation. The dependence of the spot size on the maximum radius deviation Δr_{max} by this mode is shown by the line with triangular markers in Fig. S1(b). All the other calculation parameters are the same as those in the main body of the article. The markers on the line are the calculated points and the value of each point is the average of 10 calculations. Clearly, the FWHM of the generated spot increases as the increasing of Δr_{max}. When $\Delta r_{max} = 50$ nm, the FWHM of the generated spot is 0.437λ which is enlarged by 1.7% than the spot of 0.43λ. When Δr_{max} of mode 1 is larger than 50 nm, the spot size enlarges rapidly.

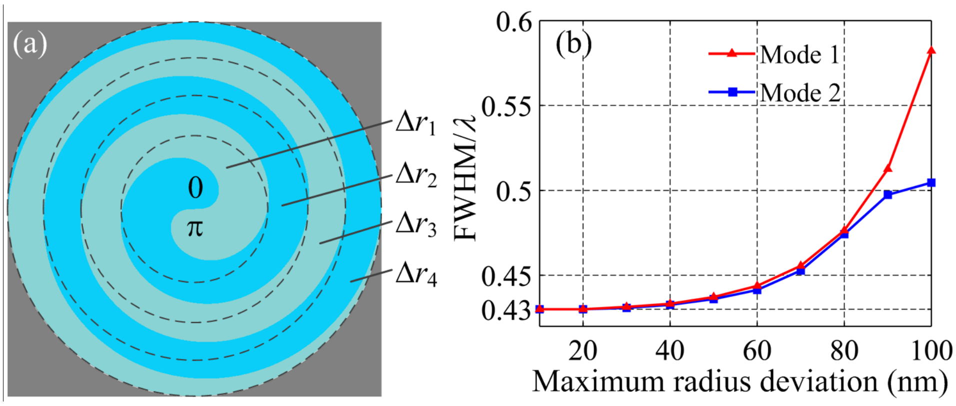

Figure S1. The dependence of the spot size on the maximum radius deviation. (a) Schematic of dividing a SZP of N_{max} into $2N_{max}$+2 zones. The radial coordinate r in each zone is added a random radius deviation Δr_i, where i ranges from 1 to $2N_{max}$+2. In this example, N_{max} = 1 and i = 1, 2, 3, 4. (b) The lateral FWHMs of the generated spots versus the maximum radius deviation for different deviation modes. Mode 1 is the deviation introduction mode that the radial coordinate of every sample point is added a random radius deviation. Mode 2 is the deviation introduction mode as shown in (a).

The second deviation mode is similar to the method for a circularly symmetrical device. The SZP of N_{max} is divided into $2N_{max}$+2 zones, as shown in Fig. S1(a). The radius of each zone equals the radius of each concentric belt of the FZP which has the same NA and N_{max} as the SZP. The radial coordinate r in each zone is added a random radius deviation Δr_i, where i ranges from 1 to $2N_{max}$+2. Fig. S1(a) shows an example of dividing a SZP of N_{max} = 1 into $2N_{max}$+2 = 4 zones. The added random radius deviation Δr_i for each zone obeys the uniform distribution with $\pm\Delta r_{max}$, where Δr_{max} is the maximum radius deviation. The dependence of the spot size on the maximum radius deviation Δr_{max} by mode 2 is shown by the line with square markers in Fig. S1(b). The markers on the line are the calculated points and the value of each point is the average of 10 calculations. The variation of the spot size along with the radius deviation of mode 2 is smaller than mode 1. When Δr_{max} = 50 nm, the lateral FWHM of the generated spot is 0.436λ which is enlarged by 1.4% than the spot of 0.43λ. Similar to the results of mode 1, the spot size enlarges rapidly when Δr_{max} is larger than 50 nm. It is reasonable to choose 50 nm as the largest tolerated fabrication deviation of the radius.

For a commercial electron beam lithography system, this tolerance 50 nm is relatively easy to achieve. For example, the minimum feature size that the EBPG5200 of Raith GmbH can achieve is less than 8nm. Therefore, the generated sub-diffraction spot is insensitive to the fabrication errors of the SZP.
